# Multimodal Characterization of Atrial Fibrillation: From Patient-Specific Anatomy and Electrophysiology to Standardized Atrial Mapping

**DOI:** 10.3390/tomography12090132

**Published:** 2026-09-14

**Authors:** Tiantian Wang, Quan Zou, Huan Yang

**Affiliations:** 1 Institute of Fundamental and Frontier Sciences, University of Electronic Science and Technology of China, Chengdu 611731, China; tiantian211231@163.com (T.W.); zouquan@nclab.net (Q.Z.); 2 Yangtze Delta Region Institute (Quzhou), University of Electronic Science and Technology of China, Quzhou 324003, China

**Keywords:** atrial fibrillation, electrocardiographic imaging, computational modeling, body surface potential mapping, standardized atrial regionalization, multimodal image fusion

## Abstract

This narrative review synthesizes a comprehensive multimodal characterization framework for atrial fibrillation (AF), tracing its progression from patient-specific anatomical reconstruction to electrophysiological phenotyping and standardized spatial mapping. The literature was identified through searches of PubMed, Web of Science, and Google Scholar, covering publications from January 2010 to June 2026, with additional studies identified from relevant references. Anatomical characterization leverages clinical imaging modalities alongside advanced deep learning models to execute precise whole-chamber, subregional, and tissue-level modeling. Electrophysiological profiling spans multi-scale modalities, including 12-lead electrocardiography (ECG), body surface potential mapping (BSPM), electrocardiographic imaging (ECGI), and electroanatomical mapping (EAM), complemented by wearable sensors for longitudinal rhythm surveillance. To bridge heterogeneous datasets across subjects and modalities, standardized coordinate systems and multimodal registration enable reproducible data integration. These integrated data parameterize patient-specific computational models to advance mechanistic insight, risk stratification, and personalized AF management. However, broad clinical translation remains constrained by imaging variability, reconstruction noise, registration uncertainty, and limited prospective multicenter validation. Future progress hinges on moving beyond technical accuracy toward prospective trials evaluating algorithm-guided clinical utility.

## Simple Summary

Atrial fibrillation (AF) is a common arrhythmia driven by structural and electrical remodeling of the atria. This review examines how combining patient-specific cardiac imaging, multi-scale electrical mapping, and standardized atrial models can improve our understanding of AF. These approaches may help identify arrhythmogenic substrates, guide catheter ablation, and support individualized treatment. The summary also highlights current challenges in data integration and encourages future tools that connect structural imaging with functional mapping for better patient care.

## 1. Introduction

Atrial fibrillation (AF) represents a major global health challenge due to its high prevalence, substantial mortality and morbidity, and heavy economic burden [1]. Its development is frequently driven by underlying atrial cardiomyopathy (AtCM), which encompasses structural, contractile, and electrophysiological abnormalities. Recurrent AF may in turn promote further atrial remodeling, resulting in a dynamic interaction between the arrhythmia and its underlying substrate [2]. Given this complexity, the 2024 ESC guidelines for AF management, built around the AF-CARE framework (Comorbidity and risk factor management, Avoid stroke and thromboembolism, Reduce symptoms by rate and rhythm control, Evaluation and dynamic reassessment), emphasize that AF is not a uniform disease but requires individualized evaluation and dynamic reassessment [3]. However, translating these principles into clinical practice remains challenging due to substantial inter-patient substrate variability [4]. Therefore, achieving precise AF phenotyping demands the seamless integration of atrial geometry, tissue architecture, mechanical function, and electrophysiological properties.

**Figure 1:**
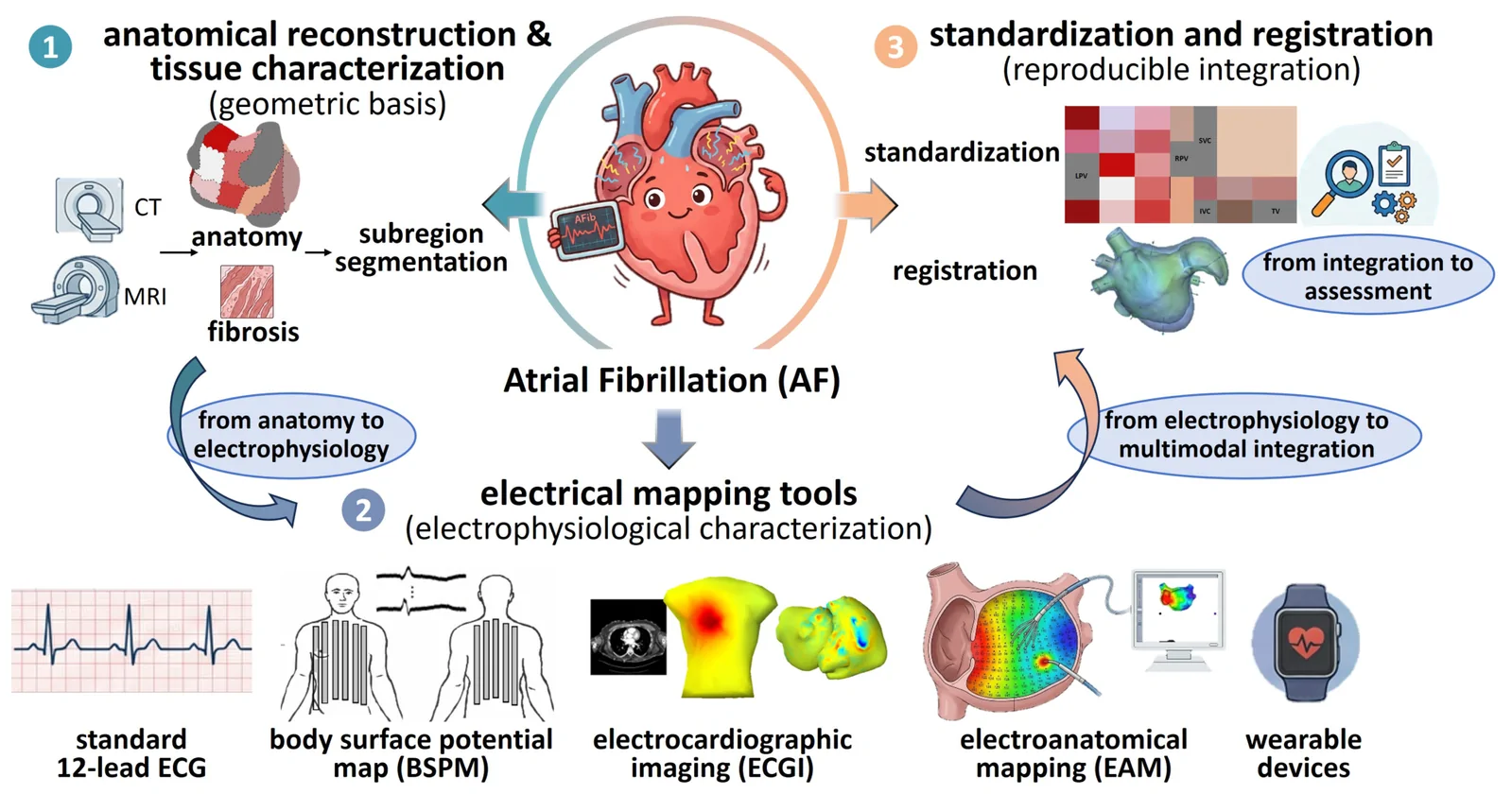
Conceptual framework for imaging-informed atrial modeling in AF. The schematic is structured around three interconnected components: (1) atrial anatomies and tissue characterization, providing the geometric basis; (2) multi-scale electrical mapping tools, characterizing atrial electrophysiology; and (3) standardization and registration, enabling reproducible cross-modality comparison and visualization.

In this narrative review, we frame atrial characterization as a progression from patient-specific anatomical reconstruction to electrophysiological phenotyping, and finally to standardized representation (Figure 1). We first discuss imaging-based segmentation and reconstruction as the basis for defining patient-specific atrial geometry and related structural features. We then examine electrical mapping across spatial scales, from surface electrocardiography (ECG) and body surface potential mapping (BSPM) to electrocardiographic imaging (ECGI) and invasive electroanatomical mapping (EAM), together with wearable approaches that provide complementary longitudinal information. Finally, we discuss standardized atrial coordinates and registration as approaches for integrating heterogeneous measurements and enabling group-level comparison. Through this framework, the review focuses on how multimodal characterization may contribute to a more comprehensive representation of atrial cardiomyopathy, thereby advancing individual AF assessment and management.

This review is intended for electrophysiologists, cardiac imaging specialists, biomedical engineers, and computational scientists, providing a structured perspective on how emerging tools may inform individual AF assessment and therapy.

## 2. Literature Search and Study Selection

Searches were performed across PubMed, Web of Science, and Google Scholar for English-language publications from January 2010 to June 2026. Search terms covered eight major areas: atrial anatomy and segmentation, atrial characterization, electrophysiological mapping (including BSPM and ECGI), electroanatomical mapping, wearable arrhythmia monitoring, multimodal registration, atrial standardization, and atlas-based mapping. Reference lists of key papers were manually screened, and citation tracking was used to identify foundational and more recent related work. AI-assisted tools supported initial abstract screening and general information synthesis. All final decisions on study inclusion and citation were made by the authors.

## 3. Anatomical Reconstruction and Tissue Characterization

Segmentation provides the geometric foundation for quantitative analysis and multimodal registration, whereas tissue and functional characterization adds information about the severity and distribution of atrial remodeling. These complementary measurements provide a structural context for understanding the heterogeneous substrate of AF and for integrating anatomical information with electrophysiological mapping. This section first discusses whole-chamber and subregional segmentation, followed by imaging-based characterization of atrial tissue and function.

### 3.1. Atrial Whole-Chamber and Subregion Segmentation

Whole-chamber segmentation can support interventional procedures by providing a patient-specific representation of atrial anatomy. In substrate-guided ablation, accurate global modeling enables volumetric and geometric measurements essential for mapping low-voltage zones and atrial fibrosis burdens [5]. Integrating these pre-procedural 3D reconstructions with intra-procedural EAM systems allows electrophysiologists to target arrhythmogenic substrates more effectively [6]. In repeat ablation procedures, whole-chamber segmentation also facilitates the identification of anatomical remodeling [7], structural gaps [8], or incomplete previous lesion sets [9], thereby minimizing procedure time and improving acute and long-term success rates. Computed tomography (CT) and magnetic resonance imaging (MRI) provide the foundational anatomical framework to define patient-specific atrial geometry. However, accurately extracting this geometry requires precise segmentation of the entire atrial structure from complex volumetric scan data. Conventional manual and semi-automated segmentation methods such as region-growing and level-sets-based methods [10] remain prevalent in clinical settings. However, these traditional approaches are time-consuming, labor-intensive, and subject to inter-observer variability [11]. The user intervention may perform poorly when atrial boundaries are indistinct, especially in LGE-MRI data affected by low contrast or artifacts [12]. To overcome these limitations, convolutional neural networks (CNNs), particularly U-Net and its variants, have become the mainstream approach for whole-chamber segmentation [13]. More recent advances incorporate multi-scale feature fusion [14] and attention mechanisms [15] to enhance spatial continuity and computational efficiency.

Beyond whole-chamber delineation, accurate pulmonary veins (PVs) segmentation is important for pulmonary vein isolation (PVI) planning. Detailed subregional segmentation enables clinicians to assess the location and morphology of the PV ostia [16], recognize anatomical variations such as common ostia and supernumerary veins [17], and guide the placement of wide circumferential ablation lines [18]. Accurate delineation of PV anatomy may also help reduce the risk of PV stenosis and incomplete isolation associated with unrecognized anatomical variations [19]. Segmenting PVs poses significant challenges due to the small diameter, complex branching topology, and substantial inter-subject variability characteristic of PVs [20,21]. To address these issues, current deep learning (DL) models typically incorporate anatomical priors or multi-kernel mechanisms [22] to achieve consistent labeling.

Furthermore, in clinical practices, precise left atrial appendage (LAA) segmentation plays a pivotal role in guiding left atrial appendage closure (LAAC). Accurate 3D characterization of LAA morphology, including landing zone dimensions and depth, facilitates optimal device sizing and selection [23]. Automated segmentation pipelines further help reduce the incidence of procedural complications such as cardiac perforation and device embolization [24]. However, the LAA exhibits substantial inter-patient anatomical variation, with phenotypes such as Chicken Wing and Cauliflower directly influencing local flow dynamics and thromboembolic risk [25]. To address these challenges, contemporary DL frameworks have integrated attention mechanisms [26], deformable convolutions [27], and latent-space or shape-prior constraints [28] to preserve 3D spatial continuity and more faithfully capture the intricate LAA geometry.

Regarding multi-regions segmentation, manual methods range from rough region partitioning [29,30] to finer multi region schemes [31,32]. Semi-automated approaches typically rely on shortest-path or template-based strategies with manually selected landmarks to segment the LA or both atria into multi regions [33,34,35,36]. To reduce labor and variability, recent automated methods have further enabled automatic landmark detection and fully automated the whole bi-atrial segmentation [37,38].

### 3.2. Tissue and Functional Characterization

Atrial remodeling in AF extends beyond changes in chamber size and geometry. The extent and distribution of fibrosis have been associated with AF substrate and investigated in relation to recurrence after catheter ablation [39]. LGE-MRI has been widely investigated for the assessment of atrial fibrosis and scars and may provide complementary information on structural remodeling [40]. However, its widespread clinical implementation faces significant challenges, including notable variability in fibrosis quantification, a lack of standardized signal intensity thresholds [41], and poor inter-center reproducibility [42]. Moreover, the relationship between imaging-defined fibrosis and electroanatomically defined scarring is not always consistent [43]. LGE-MRI should therefore be regarded as a complementary marker of structural remodeling rather than a direct surrogate for the electrophysiological substrate. Building a comprehensive structural profile also requires evaluating fine-scale anatomical features like atrial wall thickness and regional morphology. However, accurately quantifying these properties remains difficult because of the thin atrial wall and limited contrast resolution in current clinical imaging [44].

Atrial structural disease is also reflected in mechanical function. Contemporary phenotyping increasingly incorporates speckle-tracking echocardiography-derived left atrial strain [45], left atrial reservoir function [46], and comprehensive atrial mechanical assessment [47]. These functional parameters can detect impaired atrial compliance and mechanical dysfunction beyond conventional volume-based assessment. By quantifying phasic atrial mechanics, these techniques capture atrial stiffness and myocardial compliance at a preclinical stage, thereby refining risk stratification beyond conventional size-based parameters [48]. Together, atrial volume, tissue characteristics, wall morphology, and mechanical function provide complementary information to atrial geometry and may improve characterization of the heterogeneous AF substrate [49]. Their incremental value for treatment selection, however, remains to be established.

### 3.3. Linking Anatomical Reconstruction to Computational Modeling and Simulation

Patient-specific atrial geometries derived from segmentation can provide the anatomical foundation for in silico electrophysiological modeling [5]. Segmented meshes can be annotated with tissue characteristics to define heterogeneous substrate properties that drive simulated activation patterns. These parameterized geometries bridge image-based phenotyping with model-driven mechanistic insight.

## 4. Electrophysiological Characterization Across Scales

**Figure 2:**
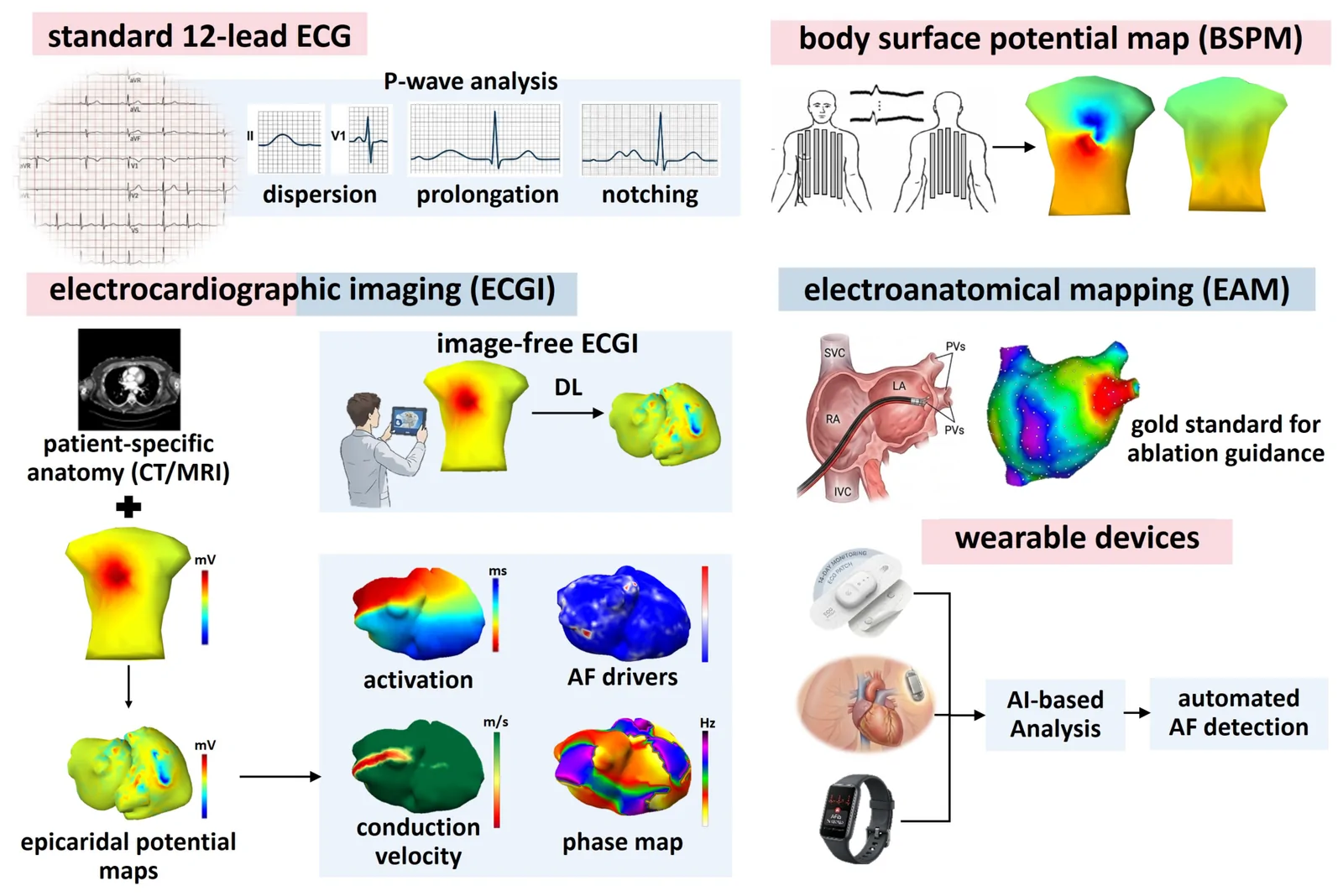
Overview of current modalities for cardiac electrical monitoring and mapping. The top panels illustrate standard 12-lead ECG, highlighting detailed P-wave analysis, alongside body surface potential mapping (BSPM). The left section details electrocardiographic imaging (ECGI), which uses BSPM and patient-specific anatomy (CT/MRI) to reconstruct epicardial potentials and compute activation, conduction velocity, AF drivers, and phase maps. An alternative imageless ECGI approach utilizing DL is also included. On the right, invasive electroanatomical mapping (EAM) is shown as the gold standard for ablation guidance, along with various wearable devices using AI-based analysis for automated AF detection. Pink = diagnostic modalities; blue = therapeutic guidance tools. ECGI is rendered in a blended color to indicate its overlap across both domains.

Electrical remodeling is a central component of the AF substrate and can be assessed at different spatial and temporal scales. Surface ECG and BSPM capture whole-heart electrical activity from the body surface. ECGI reconstructs these signals back to the atrial surface. EAM provides high spatial resolution through direct endocardial contact. Wearable monitors extend the temporal dimension outside the clinic. Spanning a spectrum from broad population-level screening to detailed, patient-specific substrate mapping, these complementary modalities yield crucial electrophysiological data to guide and validate computational atrial models. Figure 2 visualizes different electrical mapping techniques.

### 4.1. Standard ECG and P-Wave Analysis

The standard 12-lead ECG serves as the fundamental reference for assessing cardiac electrical activity in clinical arrhythmia diagnosis [50], with P-wave morphology offering a direct window into atrial substrate remodeling. Standard parameters such as P-wave prolongation reflect delayed atrial conduction [51], whereas P-wave notching may indicate atrial enlargement [52]. Beyond these conventional parameters, a broader spectrum of clinically validated P-wave markers provides critical insight into atrial substrate remodeling. Advanced interatrial block (AIAB) strongly correlates with left atrial dysfunction and incident AF [53], whereas an abnormal P-wave terminal force in lead V1 serves as a well-established surrogate for left atrial overload and electromechanical delay [54]. Likewise, shifts in the P-wave axis capture spatial depolarization abnormalities [51], and high-gain amplified P-wave analysis enables fine-grained detection of subtle intra-atrial conduction delays [55].

To capture subtle electrophysiological signals beyond visual inspection, DL models have been increasingly adopted. While some models focus on immediate AF detection from brief tracings [56,57], others target predictive tasks such as forecasting incident AF [58], estimating post-ablation recurrence risk [59], or identifying subclinical atrial myopathy [60]. Although conventional ECG provides limited spatial information [61], these surface markers can capture clinically relevant manifestations of the atrial substrate and remain important for routine assessment.

### 4.2. Body Surface Potential Mapping

BSPM extends the standard 12-lead ECG using a dense electrode array (64–256 electrodes) to record electrical activity over the thorax. This extensive spatial coverage provides a detailed representation of the distribution of cardiac potentials [62]. In the context of AF, BSPM enables the non-invasive localization of focal sources and dominant rotors that sustain the arrhythmia [63,64]. Furthermore, BSPM helps characterize atrial depolarization patterns and elucidate the underlying mechanisms of AF maintenance [65]. Studies have also examined how many electrodes are needed for effective mapping, particularly for identifying AF drivers, suggesting that optimized electrode placement may preserve diagnostic value while reducing system complexity [66]. Although BSPM continues to offer valuable electrophysiological insights in research settings, it has not yet been incorporated into routine, guideline-directed patient management or standard diagnostic workflows [67].

### 4.3. Electrocardiographic Imaging (ECGI)

ECGI is a non-invasive technique that combines patient-specific cardiac anatomy with body surface potentials to reconstruct electrical activity on the heart surface [68]. In AF, ECGI has been used to identify and characterize key electrophysiological features, such as drivers [69], rotors [70] and zones of slow conduction [71], making it useful for pre-procedural planning, substrate assessment, and guiding targeted ablation. It can also distinguish paroxysmal from persistent AF and provide insights into atrial electrical remodeling [69]. However, current evidence regarding ECGI-guided ablation remains limited, heterogeneous, and largely exploratory [72], with its definitive superiority over standard ablation strategies yet to be established in robust clinical trials [73]. In addition, conventional ECGI relies on CT or MRI for anatomical modeling, with CT involving radiation exposure and MRI being relatively time-consuming. Novel imageless ECGI techniques without CT/MRI have therefore attracted growing interest. These techniques yield robust estimates of activation rates [74], as well as real-time assessment of atrial substrate heterogeneity [75] and reconstruction of atrial surface potentials [76].

Despite these advances, broad translational adoption of ECGI remains constrained by several technical and validation bottlenecks. The reconstructed electrical activity is sensitive to measurement noise, respiratory motion artifacts, signal preprocessing, electrode localization, torso-cardiac geometry, anatomical segmentation, regularization, and assumptions regarding the underlying cardiac source model [77,78,79,80]. Differences in acquisition protocols and reconstruction algorithms further complicate comparisons across studies. Coupled with a lack of extensive multicenter clinical validation, these limitations underscore that ECGI should currently be viewed as an evolving investigational methodology rather than a proven diagnostic standard [78].

### 4.4. Electroanatomical and High-Density Mapping

Invasive EAM remains the clinical gold standard for guiding catheter ablation of complex arrhythmias. Modern high-density EAM systems provide detailed spatial information and enable precise characterization of complex activation patterns, including slow conduction zones [81], re-entrant circuits [82], and focal drivers that sustain AF [83]. Technically, these capabilities have been augmented by advanced mapping algorithms. Omnipolar mapping facilitates orientation-independent signal resolution [84], coherent mapping reconstructs vector-based propagation patterns [85], and ripple mapping allows threshold-free visualization of wave-front dynamics [86]. When integrated with local conduction velocity mapping, these tools permit precise delineation of pathological slow conduction and have further served as an invasive reference to validate non-invasive ECGI techniques [79].

Nevertheless, EAM is time-consuming and technically demanding [87], which has motivated efforts to automate parts of the analysis workflow [88]. In addition, challenges remain in capturing the global electrical activity of the atria and in mapping unstable AF [89]. This is particularly evident in the ongoing controversy surrounding the identification of AF drivers and rotors, where different mapping modalities have yielded inconsistent and sometimes unrepeatable results across clinical studies [90,91].

### 4.5. Wearable and Ambulatory Rhythm Monitoring

Wearable and ambulatory rhythm monitoring has changed arrhythmia diagnosis from brief, clinic-based recordings to continuous, real-world surveillance. This is especially valuable for AF, which is often paroxysmal and asymptomatic, and can be missed even on standard Holter monitoring [92].

Prolonged patch-based monitoring can substantially improve diagnostic yield. For example, 14-day ECG patches identify far more arrhythmias than 24 h recordings in patients with ischemic stroke or syncope [93], making them particularly useful for capturing transient AF episodes that would otherwise go undetected. For longer-term follow-up, implantable loop recorders (ILRs) provide high-quality monitoring over months to years and are well suited for AF detection [94]. Beyond clinical devices, consumer wearables such as smartwatches with photoplethysmography (PPG) [95] or single-lead ECG [96] have made large-scale opportunistic screening increasingly feasible. These advances are further amplified by AI-driven algorithms that automate AF detection with low computational overhead [97,98]. However, rhythm detection must be distinguished from clinical management. Identifying AF does not automatically improve patient outcomes unless combined with evidence-based management strategies [3].

### 4.6. Linking Electrical Mapping to Validation

Multi-scale electrical mapping plays a pivotal role in personalizing computational atrial models by capturing both spatial mechanisms and temporal dynamics. On the spatial level, high-resolution data from invasive EAM and non-invasive ECGI or BSPM are mapped onto patient-specific geometries to constrain local tissue properties and parameterize the model. On the temporal level, longitudinal rhythm annotations from wearable and ambulatory monitors track AF burden and disease recurrence over time, providing essential long-term benchmarks to calibrate and validate model predictions of arrhythmia progression and treatment response. Integrating these complementary spatial and temporal dimensions allows computational models to move beyond generic representations toward true patient-specific electrophysiological characterization.

## 5. Standardization and Registration

Patient-specific characterization is informative only if measurements obtained from different modalities can be related to one another and compared across individuals. Atrial size, shape, PV anatomy, and regional boundaries vary substantially between individuals [99], while imaging and electrophysiological measurements are acquired using different coordinate systems and spatial resolutions. Standardization and registration bridge this gap by establishing a common spatial reference, enabling the mapping of structural and electrical abnormalities onto a unified coordinate system. Table 1 summarizes the studies of atlas-based atrial mapping.

### 5.1. Standardized Atrial Coordinates and Regionalization

Standardized coordinates provide a common spatial framework for representing, quantifying, and comparing patient-specific atrial data. One of the most established approaches is the Universal Atrial Coordinate (UAC) system [100]. By converting irregular atrial surfaces into standardized maps, this system provides a basis for evaluating structural and electrical features in a uniform domain [101].

To complement 3D representations, which require manual manipulation on 2D screens for complete view coverage [102], standardized 2D surface unfolding offers an intuitive visual representation of complex atrial geometries. Early approaches focused mainly on flattening the left atrium (LA) for procedural visualization [103,104], whereas later methods incorporated more consistent anatomical organization to facilitate comparison between subjects [105]. These approaches have subsequently been extended to the right atrium (RA) and to bi-atrial representations [35,36,100,106]. Although planar projections inevitably introduce spatial distortion, their primary strength lies in providing an immediate, standardized global view rather than preserving absolute metric distances.

Regionalization further defines anatomically meaningful segments across whole-atrial geometries. Landmark- and rule-based methods, atlas registration, and more recent automated approaches have been used to establish reproducible LA and RA regions [32,33,34,35]. Recent consensus work has further emphasized standardized atrial regionalization for 3D electroanatomical mapping, cardiac imaging, and computational modeling [107]. The importance of regionalization is not simply to divide the atrium into predefined segments, but to provide consistent anatomical labels for describing where structural and electrical abnormalities occur.

### 5.2. Multimodal Registration and Fusion

While standardization establishes a population-wide reference framework, multimodal registration focuses on spatial alignment within individual datasets. In AF, this may involve aligning atrial anatomy with functional measurements.

Traditional intensity-based methods remain widely used in clinical settings. For CT and MRI, mutual information (MI) or normalized mutual information (NMI) is the standard similarity measure because it does not require direct intensity matching [108]. They are often combined with rigid or affine pre-alignment followed by deformable optimization. Feature-based methods offer an alternative when intensity distributions differ drastically. These approaches rely on selected landmarks to establish spatial correspondence [109]. Surface-based iterative closest point (ICP) can be used to register the geometry without selecting the landmarks [110]. However, this method is susceptible to speckle noise.

DL has introduced transformative advancements in multimodal deformable registration by enabling end-to-end prediction. Supervised atrial registration typically uses CNN-based models to predict dense displacement fields, with ground truth mostly derived from the registration of segmented anatomical shapes [111]. Since true gold-standard deformations are difficult to obtain, most recent studies rely on weakly supervised [112], while unsupervised frameworks still dominate the literature. Unsupervised registration models enable atria alignment by optimizing similarity-driven losses, avoiding ground-truth labels [113]. To address error accumulation in such learning-based pipelines, recent architectures increasingly incorporate probabilistic motion fields [114] or Bayesian estimation to quantify registration confidence [115], ensuring that uncertainty metrics can be explicitly accounted for during subsequent electrophysiological simulations.

### 5.3. Linking Standardization and Registration to Parameterization and Computational Modeling

Standardized atrial coordinates and multimodal registration provide the spatial framework to seamlessly integrate heterogeneous data into computational models. Specifically, registration techniques align anatomical imaging with electrophysiological data within an individual patient’s geometry. Moving beyond single-subject analysis, standardized coordinates map these patient-specific models onto a shared reference frame. This enables multicenter datasets to be pooled and compared without geometric normalization bias [105], improves the generalizability of DL models on uniform input domains [116], and allows reliable tracking of tissue remodeling in serial follow-up studies [117]. The integrated framework spans individual substrate characterization to population-level analyses, including the construct of virtual cohorts for investigating AF mechanisms.

## 6. AI for Integrated Diagnosis and Treatment Selection

The preceding sections describe a progression from patient-specific anatomical and structural characterization to electrophysiological assessment, followed by the standardization and integration of heterogeneous measurements. Recent comprehensive reviews have further mapped the evolving landscape of AI applications across these complementary data, highlighting both the technical promise and the remaining translational gaps [118].

In diagnostic applications, AI-assisted analysis enhances the detection of paroxysmal or subclinical AF across continuous monitoring streams and short ECG tracings. Beyond rhythm identification, AI algorithms can fuse 3D atrial geometry, structural fibrosis, and mechanical strain to characterize broader atrial cardiomyopathy phenotypes. This AI-driven integration is clinically essential because persistent stroke risk and disease progression often decouple from rhythm status alone, especially in thromboembolism [119]. Underlying atrial cardiomyopathy and mechanical dysfunction frequently persist despite successful sinus rhythm restoration.

For treatment selection, AI and computational models could potentially support decision-making across the full spectrum of AF management strategies, rather than being restricted to ablation-target selection. For rate control versus rhythm control, AI models can integrate AtCM metrics and comorbidities to help clinicians choose between these foundational approaches [120]. For antiarrhythmic drug therapy, computational tools may assist in evaluating response likelihood to optimize drug selection and dosing [121]. In the context of catheter ablation, combining pre-procedural imaging, low-voltage substrates, and prior lesion sets helps estimate benefit from ablation beyond standard target mapping [122]. For patients requiring repeat ablation, AI can aid in identifying structural gaps or incomplete previous lesion sets that may harbor recurrent arrhythmogenic substrate [123]. Additionally, multimodal assessment of atrial mechanical function and appendage morphology can refine stroke risk estimation to guide anticoagulation strategies [124].

However, it is critical to distinguish proof of technical accuracy from proof that algorithm-guided decisions improve clinical outcomes. High algorithm accuracy in segmentation, driver tracking, or outcome prediction does not inherently translate to improved patient care [118]. Demonstrating genuine clinical utility requires moving beyond retrospective model validation. Future prospective trials must evaluate whether algorithm-guided decisions alter treatment choices, lower complications, and deliver superior clinical outcomes compared to standard evidence-based practice.

## 7. Challenges and Validation

To place the discussed methods in a clinical context, Table 2 summarizes their current level of clinical readiness and the main barriers to wider adoption. The readiness levels are qualitative and reflect current clinical application and validation rather than rigid metrics.

The clinical value of imaging-informed atrial computational approaches ultimately depends on whether their performance is maintained across patients, institutions, acquisition protocols, and clinical settings. Although many methods show promising results in controlled datasets, validation remains uneven across modalities and applications.

Generalizability is particularly important for atrial segmentation, standardization, and registration. Segmentation performance can be affected by image quality, scanner characteristics, acquisition protocols, and anatomical variants [125], while atlas-based methods depend on landmark definition and registration accuracy. These sources of variability can influence downstream measurements and patient-specific model construction. Standardized regionalization and registration should therefore be validated across diverse populations and imaging modalities rather than assumed to provide a universally consistent representation [126].

Reproducibility remains another important limitation. Measurements of fibrosis, wall morphology, activation patterns, and conduction properties may vary with acquisition, preprocessing, electrode configuration, mapping density, and analysis parameters [40]. This is also relevant when EAM is used as a reference for non-invasive mapping, because EAM itself is affected rhythmic conditions, catheter type, contact force, electrode size, filtering settings, bipolar versus unipolar configurations, and mapping density [127,128,129]. Validation should consequently consider test–retest and inter-center variability in addition to agreement with a reference standard.

Multimodal integration introduces further uncertainty because anatomical, structural, electrical, and longitudinal data are acquired at different spatial and temporal scales [130]. Registration errors between CT or MRI and BSPM, ECGI, or EAM can alter the apparent location of electrical abnormalities and propagate into subsequent computational models [131,132,133]. Validation should therefore assess not only the accuracy of individual components, but also whether multimodal registration and data fusion preserve clinically relevant information.

Clinical translation remains constrained by both technical and practical factors. BSPM and ECGI require reliable acquisition and anatomical registration [134], while EAM is invasive, time-consuming, and operator-dependent [135]. Wearable monitoring can extend rhythm surveillance but still requires confirmation of positive alerts [136]. AI and computational models face additional challenges in external validation, interpretability, regulatory approval, data privacy, and integration into clinical workflows [137]. Across these technologies, reducing manual verification and acquisition burden will be essential for routine use.

Most importantly, technical accuracy does not necessarily demonstrate clinical utility. High performance in segmentation, electrical mapping, or outcome prediction does not establish that algorithm-guided decisions improve patient care [138]. Future studies should therefore progress from retrospective and single-center validation toward multicenter and prospective studies that evaluate reproducibility, workflow integration, and patient outcomes [139]. At present, most advanced computational approaches should be regarded as adjunctive or investigational technologies rather than replacements for established clinical assessment.

Beyond prospective clinical trials, bridging the gap from benchmark performance to routine clinical practice also requires navigating clear regulatory approval pathways [140], alongside establishing transparent, explainable AI models that foster clinician trust [141]. Crucially, as these approaches rely heavily on multi-site data streams, robust data privacy frameworks and privacy-preserving protocols [142,143] must be integrated to safeguard patient information.

## 8. Conclusions

The integration of computational models with cardiac imaging has reshaped how AF is studied in both research and clinical settings. Accurate anatomical segmentation, electrical mapping, and spatial normalization provide complementary information on atrial structure, electrical behavior, and remodeling. Their integration enables individualized substrate characterization that may inform diagnosis, risk assessment, and treatment selection across the spectrum of AF management. Moving forward, the field must transition from pure algorithmic advances toward clinical translation guided by modern consensus concepts. By embedding atrial cardiomyopathy metrics and standardized regionalization into computational pipelines, these modeling tools can move beyond procedural visualization toward supporting broader therapeutic decisions.

However, most computational approaches remain investigational rather than established clinical tools. Their broader adoption requires robust external and multicenter validation, prospective studies demonstrating that algorithm-guided decisions improve patient outcomes, and pragmatic solutions to workflow, regulatory, and data-privacy barriers. Future work should therefore focus not only on technical accuracy, but also on reproducibility, clinical utility, and integration into routine electrophysiological care.

## Figures and Tables

**Table 1:** Overview of standardization and registration.

Category	Method	Key Features	Validation	Advantages	Limitations	Maturity & Clinical
3D Atlases	UAC	reproducible 3D coordinate field; extended to volumetric models	cohort validation	common reference frame, cross-subject comparison	landmark variability	research
2D unfolding	LA flattening	projects LA onto 2D plane	limited validation	consistent framework; multimodal fusion	distortion; landmark variability; LA only	research
	bi-atrial maps	fixed LA, RA regional layout	multi-cohort use growing	consistent framework; multimodal fusion	distortion; landmark variability	research
multimodal registration	traditional	intensity-based, feature-based, and surface-based methods	clinically validated	robust; interpretable	noise-sensitive; computationally heavy	research, early translation
	DL-based	end-to-end; supervised, unsupervised	benchmark results promising	fast; handles complex deformations	data bias; limited interpretability	research, early translation

**Table 2:** Current technology readiness across anatomical, electrophysiological, and standardized atrial mapping approaches.

Modality	Main Clinical/Research Role	Current Technology Readiness	Main Clinical Limitations
segmentation	patient-specific anatomy; procedural planning; model construction	research; early translation	anatomical variability; image quality; inter-observer variation; limited cross-center generalizability
tissue and functional characterization	fibrosis, wall morphology, chamber mechanics, and substrate assessment	research; early translation	measurement variability; limited standardization; uncertain incremental clinical value
12-lead ECG	AF diagnosis; P-wave and and atrial conduction assessment	research; clinical practice	limited spatial information; low sensitivity to localized substrate
BSPM	high-density non-invasive electrical mapping	research; investigational	electrode placement; acquisition burden; limited workflow integration
ECGI	non-invasive atrial activity mapping	research; investigational; early translation	ill-posed inverse problem; noise; anatomical dependence; limited multicenter validation
EAM	intra-procedural mapping; substrate characterization; ablation guidance	clinical practice	invasive; time-consuming; operator-dependent; limited global coverage
wearable; ambulatory monitoring	longitudinal AF detection and rhythm surveillance	clinical; rapidly expanding	false-positive alerts; limited electrophysiological information; outcome benefit remains uncertain
standardization	visualization; regionalization	research; investigational	landmark variability; geometric distortion; limited validation across populations
registration	alignment of anatomy, structure, and electrophysiology	research; investigational; early translational	uncertainty; modality differences; error propagation; limited standardization

## Data Availability

No new data were created or analyzed in this study. Data sharing is not applicable to this article.

## Abbreviations

The following abbreviations are used in this manuscript:

AF	atrial fibrillation
AIAB	advanced interatrial block
AtCM	atrial cardiomyopathy
BSPM	body surface potential mapping
CT	computed tomography
CNN	convolutional neural network
DL	deep learning
ECG	electrocardiogram
ECGI	electrocardiographic imaging
EAM	electroanatomical mapping
ICP	iterative closest point
ILR	implantable loop recorder
LGE-MRI	late gadolinium enhancement magnetic resonance imaging
LA	left atrium
LAA	left atrial appendage
MRI	magnetic resonance imaging
MI	mutual information
NMI	normalized mutual information
PV	pulmonary vein
PPG	photoplethysmography
RA	right atrium
UAC	Universal Atrial Coordinate
